# Enhancing fracture diagnosis in pelvic X-rays by deep convolutional neural network with synthesized images from 3D-CT

**DOI:** 10.1038/s41598-024-58810-4

**Published:** 2024-04-05

**Authors:** Rashedur Rahman, Naomi Yagi, Keigo Hayashi, Akihiro Maruo, Hirotsugu Muratsu, Syoji Kobashi

**Affiliations:** 1 Graduate School of Engineering, University of Hyogo, 2167 Shosha, Himeji, 671-2201 Japan; 2 Advanced Medical Engineering Research Institute, University of Hyogo, 3-264 Kamiya-cho, Himeji, Hyogo 670-0836 Japan; 3 Hyogo Prefectural Harima-Himeji General Medical Center, 3-264 Kamiya-cho, Himeji, Hyogo 670-8560 Japan

**Keywords:** Biomedical engineering, Machine learning

## Abstract

Pelvic fractures pose significant challenges in medical diagnosis due to the complex structure of the pelvic bones. Timely diagnosis of pelvic fractures is critical to reduce complications and mortality rates. While computed tomography (CT) is highly accurate in detecting pelvic fractures, the initial diagnostic procedure usually involves pelvic X-rays (PXR). In recent years, many deep learning-based methods have been developed utilizing ImageNet-based transfer learning for diagnosing hip and pelvic fractures. However, the ImageNet dataset contains natural RGB images which are different than PXR. In this study, we proposed a two-step transfer learning approach that improved the diagnosis of pelvic fractures in PXR images. The first step involved training a deep convolutional neural network (DCNN) using synthesized PXR images derived from 3D-CT by digitally reconstructed radiographs (DRR). In the second step, the classification layers of the DCNN were fine-tuned using acquired PXR images. The performance of the proposed method was compared with the conventional ImageNet-based transfer learning method. Experimental results demonstrated that the proposed DRR-based method, using 20 synthesized PXR images for each CT, achieved superior performance with the area under the receiver operating characteristic curves (AUROCs) of 0.9327 and 0.8014 for visible and invisible fractures, respectively. The ImageNet-based method yields AUROCs of 0.8908 and 0.7308 for visible and invisible fractures, respectively.

## Introduction

Pelvic fractures, encompassing both hip fractures and pelvic ring fractures, present a challenging medical condition due to the unique shape and characteristics of the pelvic bones. Pelvic fractures can result in severe complications such as nerve damage, bladder or bowel dysfunction, and internal bleeding, resulting in increased morbidity and mortality rates. Hence, pelvic fracture can be considered as a significant health concern, particularly in older adults and those with underlying medical conditions^1^. Moreover, pelvic fracture is a leading cause of death among the elderly^2,3^. Early diagnosis of pelvic fractures is crucial for timely interventions as well as lowering the risk of mortality.

The plain pelvic radiograph, commonly referred to as pelvic X-ray (PXR), plays a crucial role in diagnosing fractures in the pelvic region. While studies have demonstrated that computed tomography (CT) has higher sensitivity and specificity in detecting pelvic fractures^4,5^, the diagnostic procedure typically starts with PXR examination^4^. However, one of the challenges associated with PXR examination is the increasing number of pelvic fractures^6–8^. This surge in cases puts additional pressure on radiologists and contributes to early misdiagnoses^9^. These misdiagnoses have negative implications, including worsened prognosis, an increase in treatment cost, and increased mortality rates. Therefore, a computer-aided diagnosis system (CAD) can help to improve the efficiency of pelvic fracture detection. Another challenge in PXR examination is the constrained viewing angle for which some fractures, especially some insufficiency fractures and osteoporotic fractures, may be invisible. Insufficiency fractures are caused by repetitive stress and some are practically invisible in PXR images^4^. Similarly, fractures associated with osteoporosis are also challenging to detect in PXR images^10^. Many osteoporosis fractures are invisible in their initial stage of development without an appropriate viewing angle. Deep learning has been demonstrated to be effective in learning subtle features and patterns to assist in different disease diagnosis^11–13^. Hence, employing a deep learning-based assistive system could prove valuable in recognizing PXR images with visible fracture, as well as invisible fracture.

In the initial stages, fracture detection methods relied on image processing techniques and computational models like morphological operations with Hough transform^14^, neighbor-conditional shape model^15^, and relaxed digital straight-line segment (RDSS)^16^. However, these methods depended on numerous parameters, and were susceptible to subject-specific limitations. Recently, deep learning has gained popularity for detecting various fractures, such as wrist fractures^17^, rib fracture^18^, femur fracture^19^, femoral neck fracture^20^, and vertebral fractures^21^. Similarly, for hip and pelvic fracture detection, methods have been proposed utilizing deep learning. Krogue et al.^22^ proposed a DenseNet-based method for detecting hip region and fracture classification from PXR images. The binary classification accuracy achieved was 93.7%, and the multi-class classification accuracy was 90.8%. Kitamura^23^ also introduced a method based on DenseNet121 model, where the model was trained to create position labeling and detect hardware presence in PXR images. A separate model was used to detect different types of fractures. The area under the curve (AUC) for position and hardware detection was 0.99. The AUCs for proximal femoral fracture, pelvic fracture, and acetabular fracture were 0.95, 0.75, and 0.85, respectively. Another method proposed the use of YOLOv4-tiny deep learning model to detect 3 types of hip fractures^24^. The model’s performance was also compared with that of doctors, achieving a sensitivity of 96.2%, while the performance of the doctors varied from 69.2 to 96.2%. The study concluded that the performance of the trained model was comparable to attending physicians and chief residents in orthopedics with no statistical difference, and outperformed the first-year residents and general practitioners. Cheng et al. proposed a scalable deep learning algorithm named PelviXNet for universal trauma detection on PXR images^25^. PelviXNet combined feature pyramid network (FPN) with DenseNet-169 and was trained using weakly supervised point annotated PXR images. The trained PelviXNet yielded an area under the receiver operating characteristic curve (AUROC) of 0.973 on a clinical population test set. All of the above methods discussed about fractures that are visible on PXR images.

Another challenge associated with deep learning is the significant amount of data required to effectively train a model. However, obtaining a substantial number of annotated medical images is often difficult. A common practice in this field is to utilize the transfer learning^26^ technique. In transfer learning, a deep learning model is initially trained on a large dataset called ImageNet^27^ for a classification task. Later, only the final layers are fine-tuned with the task-specific dataset. This approach was applied in previous studies on hip and pelvic fractures^22–25^. However, a recent study has demonstrated a more efficient three-step training scheme for transfer learning, which significantly reduced the labeled medical image requirements by 688-fold compared to the conventional two-step transfer learning, while maintaining similar performance^28^. In this proposed three-step training process, the deep learning model was first initialized with the ImageNet dataset^27^. Then, in the second step, the model was re-trained using a large chest X-ray (CXR) dataset to detect normal and abnormal cases. Finally, in the third step, the model was trained with a small dataset to detect a specific pulmonary disease. Another study utilized plain radiographs to train a deep learning model for detecting limbs, and then fine-tuned the model using PXR images for hip fracture detection^29^. The accuracy of hip fracture detection reached 91%.

A subset of deep learning, deep convolutional neural network (DCNN), has demonstrated remarkable performance across diverse applications including image classification^30,31^, object detection^32–35^, and video processing^36,37^. One of the key characteristics of DCNNs is their ability to recognize and extract features automatically, without human supervision^38,39^. This capability enables DCNNs to generate equivalent representations, facilitate sparse interactions, and implement parameter sharing^40^. As a result, different DCNNs have been used for the diagnosis and detection of various diseases^41^. Ibrahim et al. introduced a modified norm-VGG16 DCNN for the diagnosis of COVID-19 and its severity levels^42^. Inoue et al. utilized Faster-RCNN-Inception-V2-COCO DCNN to automatically detect fractures in whole-body trauma CT^43^. Ukai et al. used DCNN-based YOLOv3 to detect fractures in images extracted from multiple orientations of 3D-CT^44^. Cina et al. proposed a method that used several DCNNs for the localization of landmarks in spine radiographs^45^.

To address the lack of substantial amounts of annotated medical images to train a DCNN, this study introduces a novel two-step transfer learning approach based on digitally reconstructed radiograph (DRR). In the first step, a deep convolutional neural network (DCNN) is trained using different numbers of synthesized PXR images derived from 3D-CT by DRR. The second step involves fine-tuning the classification layers of the DCNN using acquired PXR images. Another contribution of this study is the performance evaluation of DCNN on different PXR datasets categorized based on fracture visibility. Furthermore, the performance of the proposed method is compared with the conventional ImageNet-based transfer learning method, and combinations of DRR-based method with ImageNet-based method. The proposed DRR-based method, using 20 synthesized PXR images for each CT, achieved AUROCs of 0.9327 and 0.8014 for visible and invisible fractures, respectively. The ImageNet-based method yielded AUROCs of 0.8908 and 0.7308 for visible and invisible fractures, respectively.

## Result

### Distribution of PXR dataset

**Figure 1:**
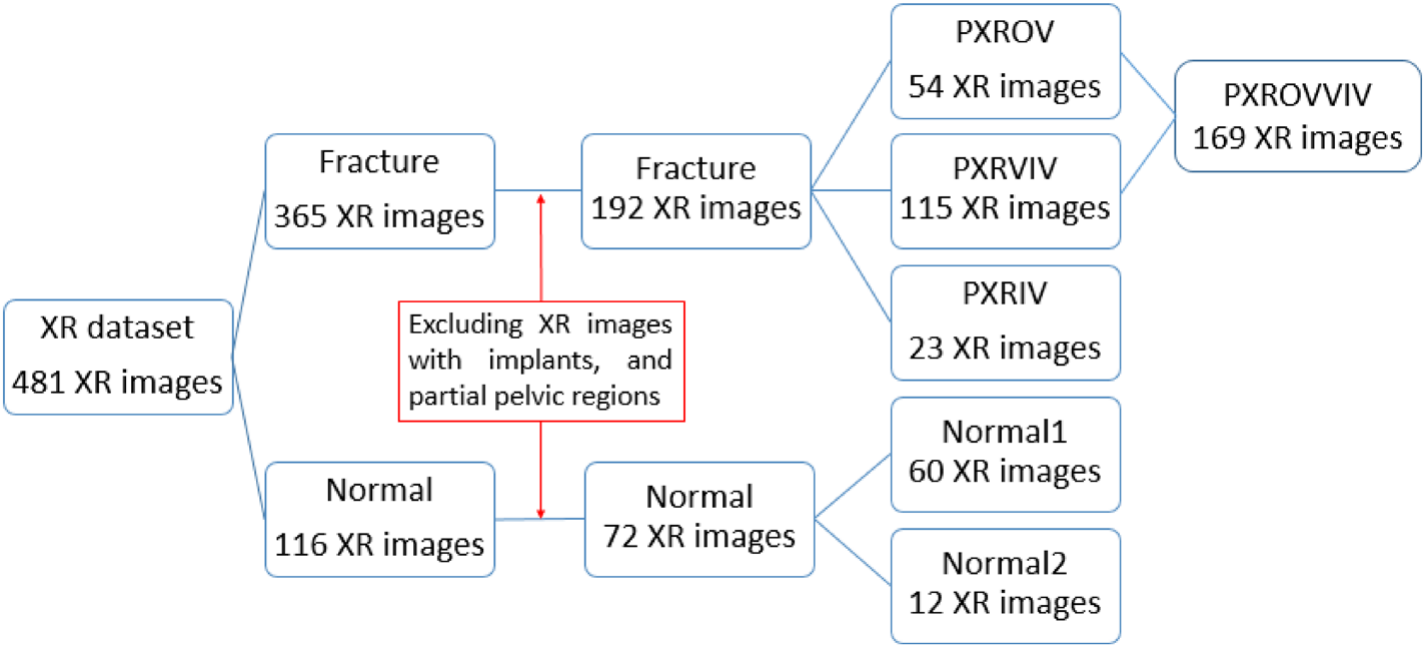
Distribution of PXR dataset.

In the PXR dataset, there were primarily two classes of images: 'fracture' class, consisting of images with fractures, and 'normal' class, comprising images without any fractures. After excluding the PXR images with implants, and partial pelvic regions, the remaining PXR images with fractures were further categorized into three groups based on the visibility of fractures: PXROV, PXRIV, and PXRVIV. PXROV included PXR images with visible fractures, PXRIV included PXR images without visible fractures but with fractures identified in the corresponding 3D-CT, and PXRVIV included PXR images with both visible and invisible fractures. Additionally, PXROV and PXRVIV were merged into a single dataset called PXROVVIV. The normal PXR images were separated into 2 groups: normal1 which contained 60 PXR images and normal2 which consisted of 12 PXR images. The normal1 group was combined with PXROV, PXRVIV, and PXROVVIV groups to assess the performance of visible fracture diagnosis. Furthermore, normal2 group was combined with PXRIV group to assess the performance of invisible fracture diagnosis. The distribution of each dataset is illustrated in Fig. 1.

### DRR-based method

**Figure 2:**
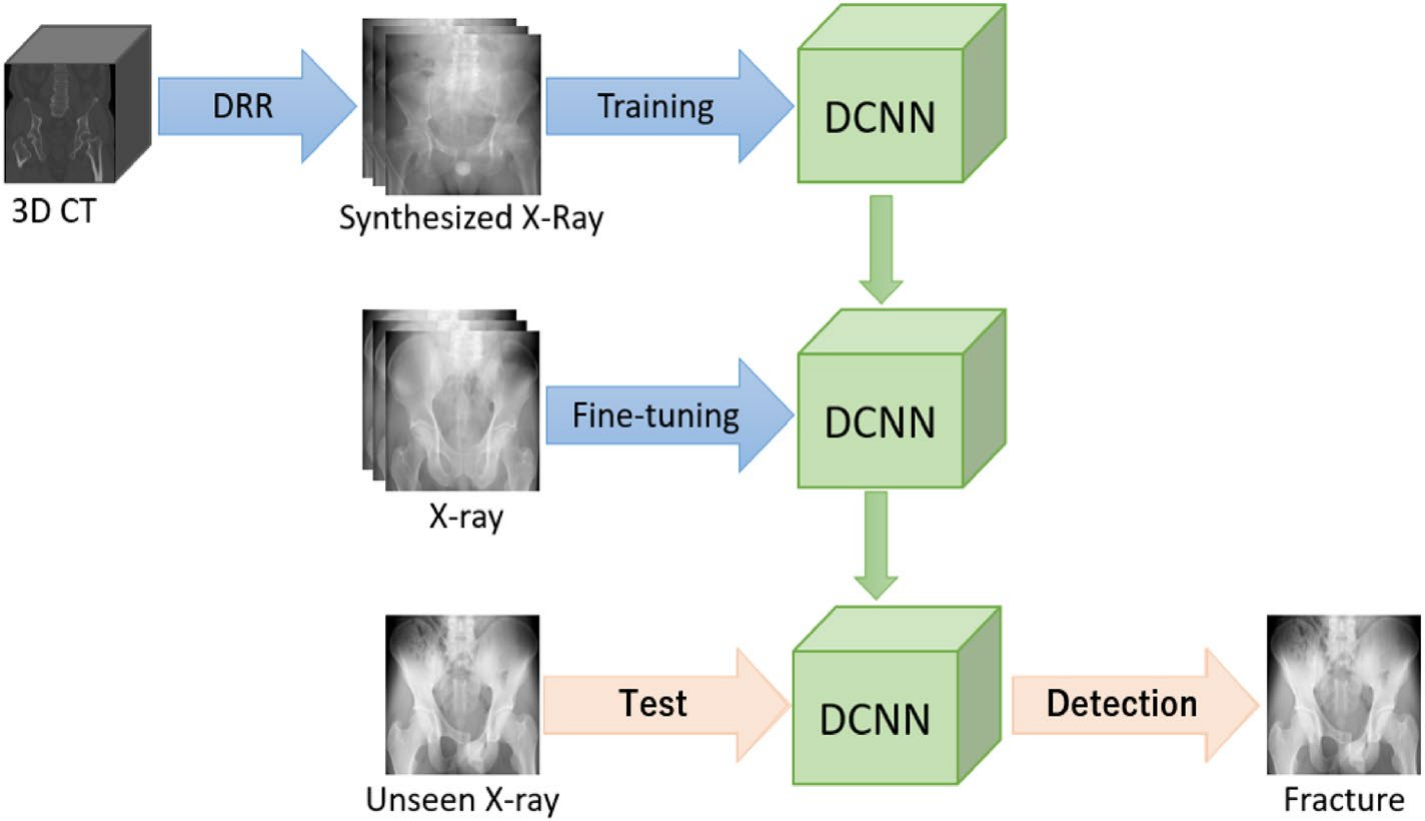
Overview of DRR-based method for PXR with fracture detection.

**Figure 3:**
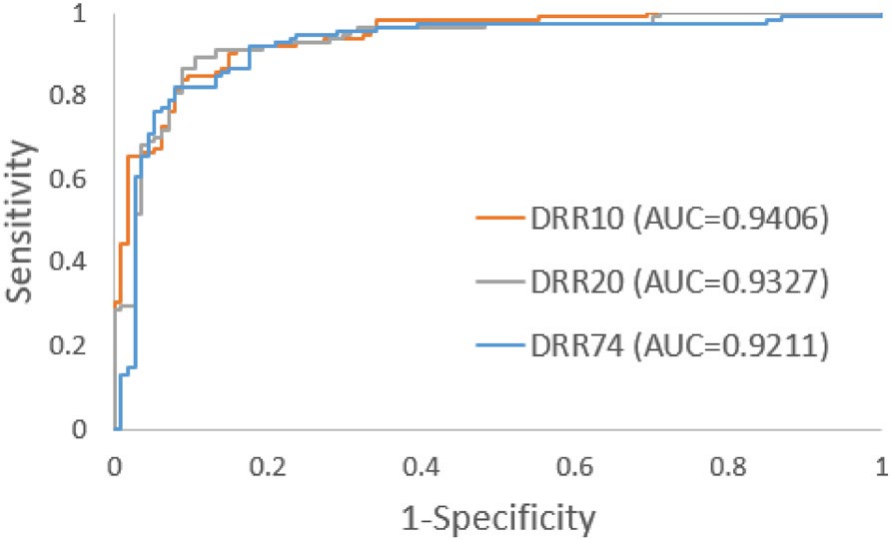
ROCs of PXROV diagnosis for DRR10, DRR20 and DRR74 training schemes.

Applying DRR on a single 3D-CT image, numerous radiographic images can be synthesized. For this study, three DRR datasets, namely DRR10, DRR20, and DRR74, were synthesized by randomly rotating the 3D-CT. Each dataset consisted of 10, 20, and 74 synthesized images, respectively, corresponding to each 3D-CT. The 3D-CT of the subjects with fractures included in the XROV, XRVIV or XRIV dataset, as well as 3D-CT with implants, were excluded. After exclusions, a total of 349 3D-CT remained, out of which 152 had fractures and 197 was normal. DRR was applied only on the pelvic region of the 3D-CT. The DCNN was trained separately using the DRR10, DRR20, and DRR74 datasets, using fivefold cross-validation. The best model for each category were selected, and only the Fully-Connected (FC), SoftMax (SM) and Classification (CL) layers were fine-tuned using the PXROV dataset. The overview of the DRR-based method is shown in Fig. 2. The area under the receiver operating characteristic (AUROC) curves for PXROV diagnosis using models trained with DRR10, DRR20, and DRR74, were 0.9406, 0.9327, and 0.9211, respectively. The ROC curves of PXROV diagnosis by models trained with DRR10, DRR20, and DRR74 are shown in Fig. 3.

**Figure 4:**
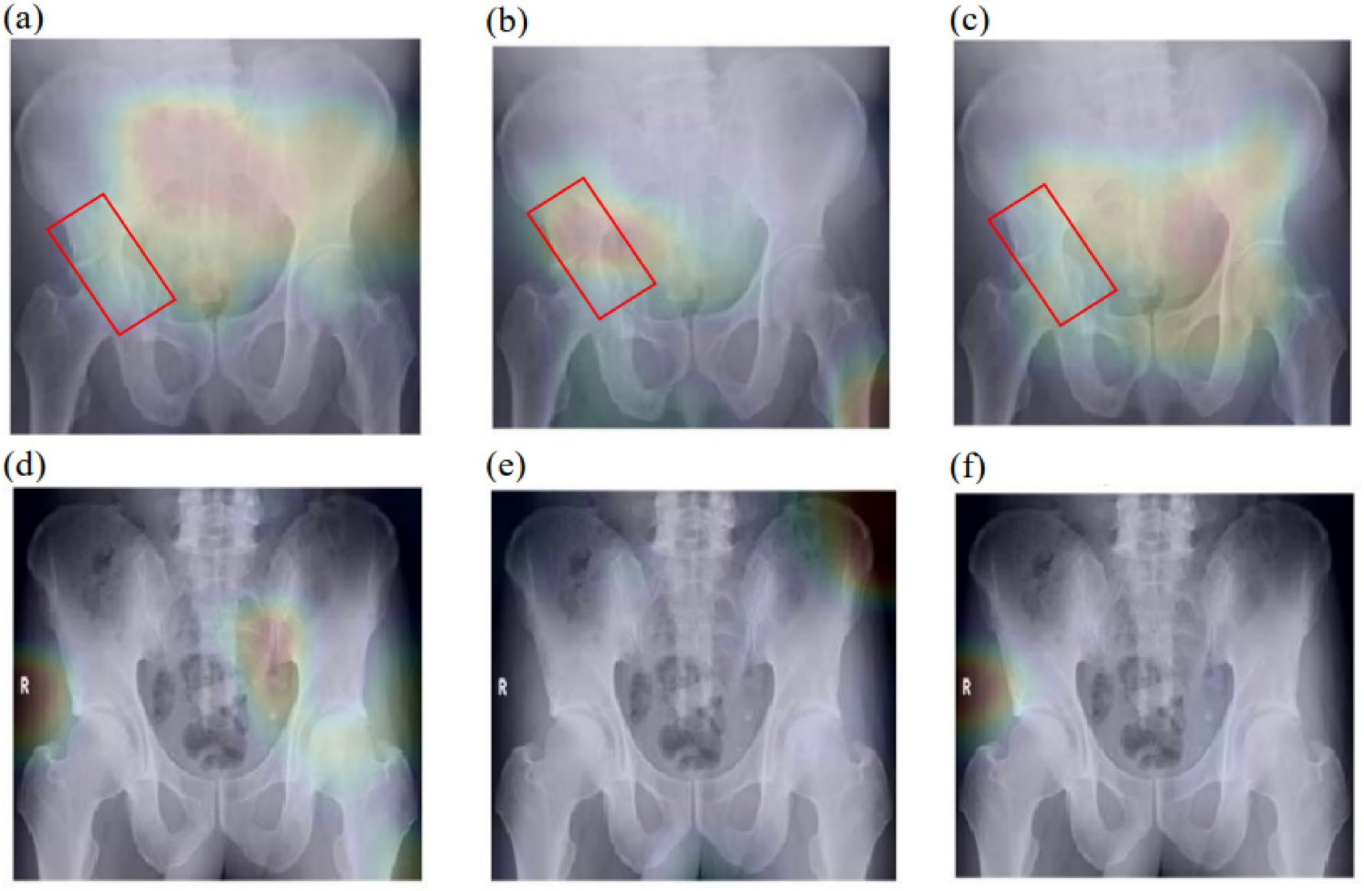
Visualization of Grad-CAM result on PXR images. (**a**) Pre-training dataset DRR10; Fracture class. (**b**) Pre-training dataset DRR20; Fracture class. (**c**) Pre-training dataset DRR74; Fracture class. (**d**) Pre-training dataset DRR10; Normal class. (**e**) Pre-training dataset DRR20; Normal class. (**f**) Pre-training dataset DRR74; Normal class.

Additionally, for the models pre-trained with DRR10, DRR20, and DRR74, the F1 scores for PXROV were found to be 0.847, 0.895, and 0.860, respectively. Hence, DRR20 was chosen for additional analysis and comparison. Grad-CAM was used to visualize the fracture region. Figure 4 shows some examples of Grad-CAM result overlaid on PXR images for visualization of relevant region.

### Comparison between DRR-based and conventional method for detecting PXR image with visible fracture

In this step, we implemented four pre-training approaches: DRR20, ImageNet, ImageNet + DRR20, and ImageNet + DRR20_Full. The ImageNet approach involved training a DCNN model initially on the ImageNet dataset, followed by fine-tuning the FC, SM, and CL layers using PXR images. In the DRR20 approach, the DCNN model was trained using the DRR20 dataset, and then the FC, SM, and CL layers were fine-tuned with PXR images. For the ImageNet + DRR20 approach, we re-trained the DCNN model pre-trained on ImageNet with the DRR20 dataset, and subsequently fine-tuned the FC, SM, and CL layers with PXR images. Lastly, in the ImageNet + DRR20_Full approach, the DCNN model pre-trained on ImageNet was first re-trained with the DRR20 dataset, and then the entire DCNN model was fine-tuned using PXR images.

**Figure 5:**
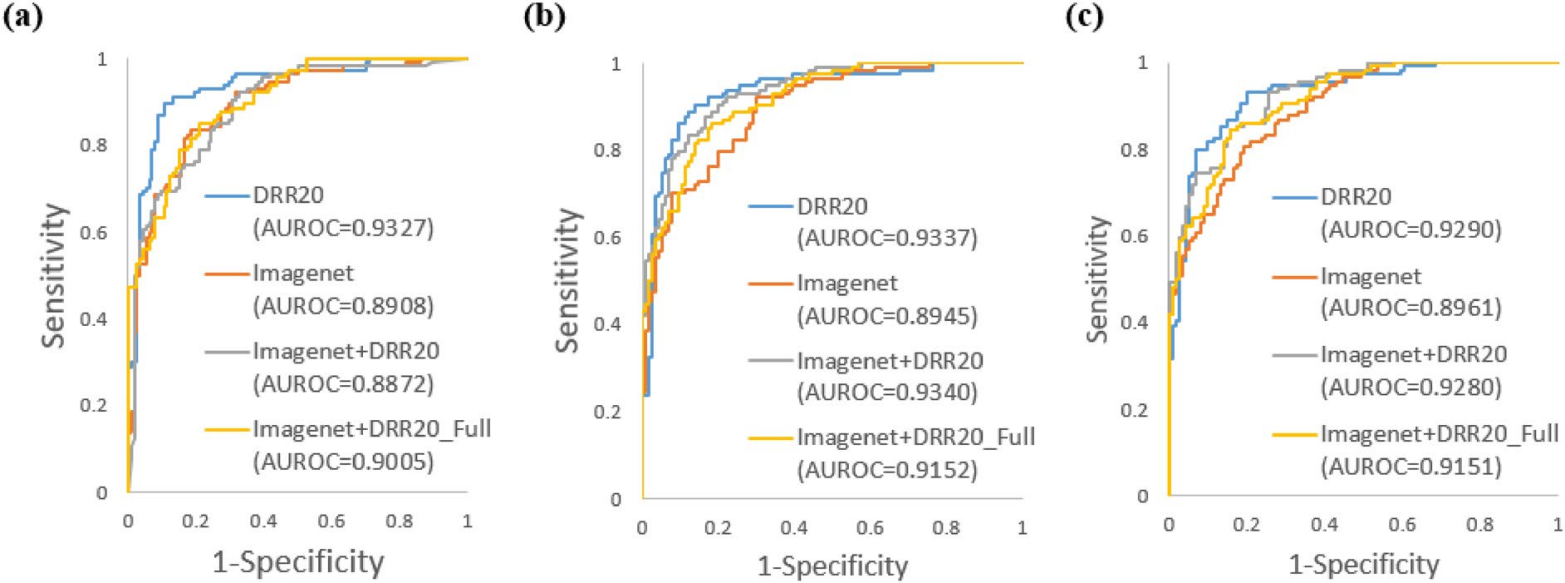
ROC curves for recognizing PXROV in DRR20, ImageNet, ImageNet + DRR20, and ImageNet + DRR20_Full training schemes. (**a**) Fine-tuning data PXROV. (**b**) Fine-tuning data PXRVIV. (**c**) Fine-tuning data PXROVVIV.

In the first evaluation, the DCNN was fine-tuned using the PXR images from the PXROV dataset, and the performance evaluation was conducted on the PXROV dataset using fivefold cross-validation. The AUROCs obtained for DRR20, ImageNet, ImageNet + DRR20, and ImageNet + DRR20_Full were 0.9327, 0.8908, 0.8872, and 0.9005, respectively. Figure 5a illustrates the corresponding ROC curves. The F1 scores achieved for DRR20, ImageNet, ImageNet + DRR20, and ImageNet + DRR20_Full were 0.895, 0.811, 0.774, and 0.804, respectively.

In the second evaluation, the PXR images from the PXRVIV dataset were utilized for fine-tuning the DCNN, and the performance evaluation was conducted on the PXROV dataset. The AUROCs obtained for DRR20, ImageNet, ImageNet + DRR20, and ImageNet + DRR20_Full were 0.9337, 0.8945, 0.934, and 0.9152, respectively. The F1 scores achieved for DRR20, ImageNet, ImageNet + DRR20, and ImageNet + DRR20_Full were 0.875, 0.800, 0.839, and 0.818, respectively. Figure 5b shows the corresponding ROC curves.

In the third evaluation, the PXROVVIV dataset was utilized to fine-tune the DCNN, and the PXROV dataset was used to assess its performance. The AUROCs obtained for DRR20, ImageNet, ImageNet + DRR20, and ImageNet + DRR20_Full were 0.9290, 0.8961, 0.9280, and 0.9151, respectively. Figure 5c displays the corresponding ROC curves. The F1 scores achieved for DRR20, ImageNet, ImageNet + DRR20, and ImageNet + DRR20_Full were 0.852, 0.800, 0.839, and 0.833, respectively.

### Comparison between DRR-based and conventional method for detecting PXR image with invisible fracture

**Figure 6:**
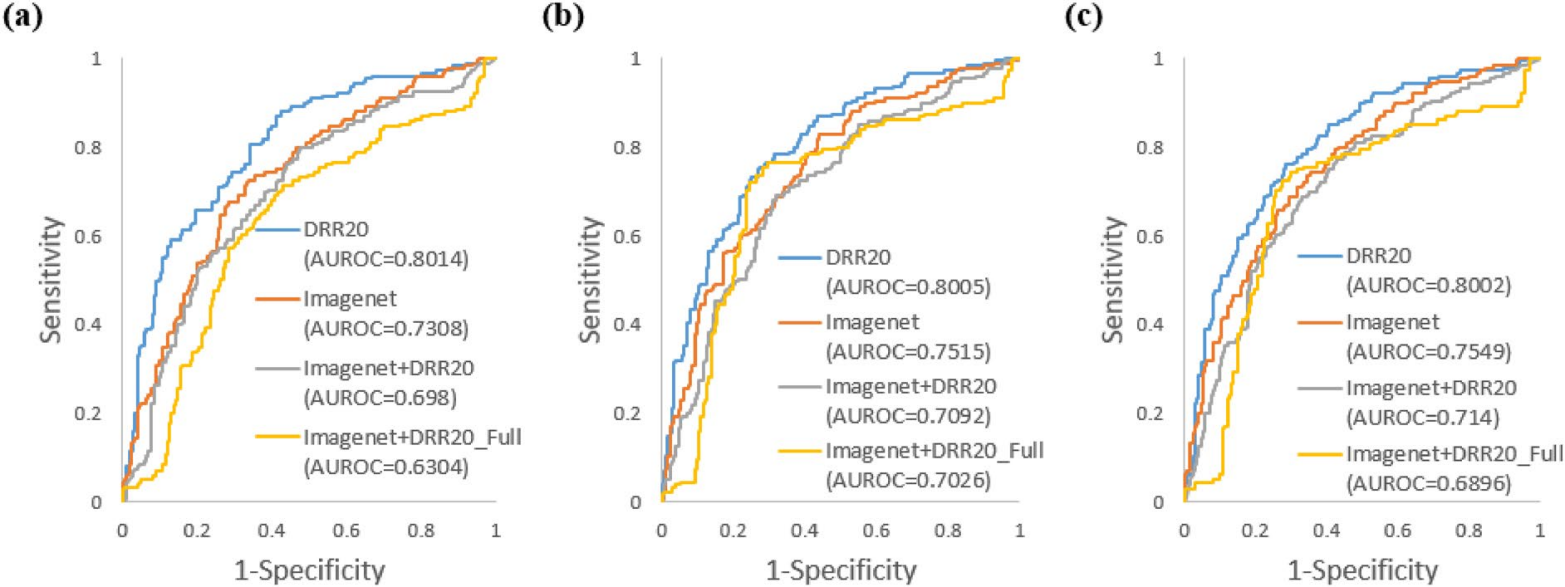
ROC curve of DCNN performance on PXRIV dataset. (**a**) Fine-tuning data PXROV. (**b**) Fine-tuning data PXRVIV. (**c**) Fine-tuning data PXROVVIV.

We have also assessed the performance of DCNNs obtained by different training schemes on the PXRIV dataset. When fine-tuning the DCNN with PXROV, the AUROCs for DRR20, ImageNet, ImageNet + DRR20, and ImageNet + DRR20_Full were found to be 0.8014, 0.7308, 0.6980, and 0.6304, respectively. Similarly, when fine-tuning the DCNN with PXRVIV, the AUROCs for DRR20, ImageNet, ImageNet + DRR20, and ImageNet + DRR20_Full were 0.8005, 0.7515, 0.7092, and 0.7026, respectively. For fine-tuning the DCNN with PXROVVIV, the AUROCs for DRR20, ImageNet, ImageNet + DRR20, and ImageNet + DRR20_Full were 0.8002, 0.7549, 0.7140, and 0.6896, respectively. The ROC curves corresponding to these results are illustrated in Fig. 6. The AUROC and F1 scores are summarized in Table 1.

**Table 1:** AUROC and F1 scores of different DCNNs on PXRIV dataset.

	Fine-tuning data PXROV		Fine-tuning data PXRVIV		Fine-tuning data PXROVVIV	
	AUROC	F1 score	AUROC	F1 score	AUROC	F1 score
DRR20	**0.8014**	**0.787**	**0.8005**	**0.792**	**0.8002**	**0.786**
ImageNet	0.7308	0.733	0.7515	0.731	0.7549	0.727
ImageNet + DRR20	0.6980	0.699	0.7092	0.726	0.7140	0.721
ImageNet + DRR20_Full	0.6304	0.692	0.7026	0.789	0.6896	0.775

Significant values are in bold.

## Discussion

In this study, our hypothesis was that pre-training a DCNN with synthesized images would enhance its performance in detecting PXR images with fractures. As DRR is a process of projecting 3D volume onto a 2D plane, the synthesized PXRs generated by random rotation contain unique anatomical variations. In contrast, conventional augmentation methods alter the locations of fractures or intensities without introducing any new anatomical variations. Hence, we proposed a DRR-based method, where the DCNN was pre-trained using synthesized PXR images generated from 3D-CT images by DRR. We also investigated the impact of the number of synthesized images on the DCNN's performance. We evaluated the AUROC for detecting PXR images with visible fractures and calculated F1 scores using a confidence score threshold of 0.5. Among 10, 20 and 74 synthesized PXR images from each 3D-CT, the AUROCs were similar for detecting PXR images with visible fractures (Fig. 3). The DCNN pre-trained with 20 synthesized PXR images achieved the highest F1 score.

Next, we compared the performance of the DRR-based method with the conventional ImageNet-based transfer learning approach, as well as combinations of both methods (Fig. 5). The summary of the results has been shown in Table 2. When detecting PXR images with visible fractures using the PXROV dataset, the DRR20 method achieved the highest AUROC and F1 score of 0.9327 and 0.895, respectively. Similarly, for the detection of PXR images with visible fractures using PXRVIV and PXROVIV datasets for fine-tuning the DCNN, the DRR20 method also achieved the highest AUROC and F1 score. Hence, irrespective of variations in the fine-tuning data based on fracture visibility, the DRR20 method outperformed ImageNet-based method. Furthermore, we explored the combination of the DRR20-based and ImageNet-based methods through ImageNet + DRR20 and ImageNet + DRR20_Full approaches. Although the AUROC values for these combinations surpassed those obtained using the ImageNet-based method, they remained lower than the DRR20-based method in almost all cases. These findings demonstrate that pre-training the DCNN with a synthesized dataset designed to the desired task enhances the learning of relevant features.

**Table 2:** AUROC and F1 scores of different DCNNs on PXROV dataset.

	Fine-tuning data PXROV		Fine-tuning data PXRVIV		Fine-tuning data PXROVVIV	
	AUROC	F1 score	AUROC	F1 score	AUROC	F1 score
DRR20	**0.9327**	**0.895**	**0.9337**	**0.875**	**0.9290**	**0.852**
ImageNet	0.8908	0.811	0.8945	0.800	0.8961	0.800
ImageNet + DRR20	0.8872	0.774	0.934	0.839	0.9280	0.839
ImageNet + DRR20_Full	0.9005	0.804	0.9152	0.818	0.9151	0.833

Significant values are in bold.

During the synthesis of PXR images using DRR, fractures that were present in the 3D-CT data were sometimes obstructed due to rotations in the 3D plane. As a result, the synthesized PXR dataset contained images with visible fractures, images without visible fractures, and normal images without any fractures. We anticipated that the trained DCNNs would capture certain unique features associated with fractures that were not visible in the images. To test this assumption, we evaluated the performance of the trained DCNNs on the PXRIV dataset. The DRR-based method demonstrated promising results in this scenario as well. Regardless of the type of fine-tuning data, DRR20 achieved the highest AUROCs (Fig. 6) and F1 scores (Table 1).

**Figure 7:**
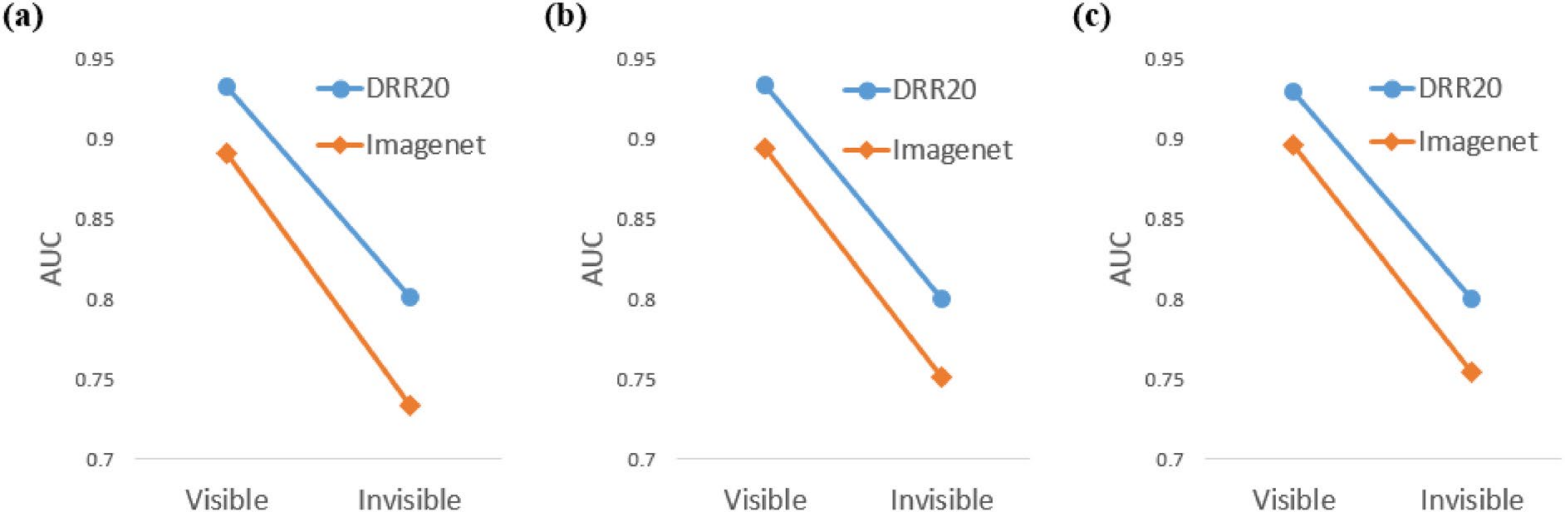
Performance comparison of visible and invisible fracture detection between DRR-based and ImageNet-based method. (**a**) Fine-tuning data PXROV. (**b**) Fine-tuning data PXRVIV. (**c**) Fine-tuning data PXROVVIV.

Although the DRR-based method achieved the highest AUROC for detecting PXR images with visible and invisible fractures, the AUROC for detecting PXR images with invisible fractures was significantly lower. This observation was also valid for the ImageNet-based method. This trend was expected since the DCNNs were not trained with the PXRIV dataset. Figure 7 illustrates the comparison of AUROCs for different fine-tuning data.

From Fig. 7a, we can see that the decrease in AUROC from detecting PXR images with visible fractures to detecting PXR images with invisible fractures was higher for ImageNet compared to DRR20 when using PXROV as the fine-tuning data. However, from Fig. 7b and c, it can be seen that the decrease in AUROC became similar for ImageNet and DRR20 when fine-tuning the DCNNs with PXRVIV and PXROVVIV datasets. This suggests that the fine-tuning dataset, which included some PXR images with invisible fractures, improved the detection of PXR images with invisible fractures. Therefore, accurate annotation of data was crucial for enhancing the performance of the DCNN in detecting PXR images with invisible fractures.

As DRR20 achieved the highest AUROC and F1 score for visible fracture (PXROV) diagnosis, we can conclude that DRR20 is the best method among DRR20, ImageNet, Imagenet + DRR20, and Imagenet + DRR20_Full. Furthermore, even though the DCNN was not optimized with PXRs that had only invisible fractures (PXRIV dataset), the DRR20 demonstrated promising AUROC and F1 score for detecting PXRs with invisible fractures. The reason for the better performance of the proposed method is that the DCNN was pre-trained using synthesized PXR images. As a result, the FC layers along with the Resnet101 backbone were specifically tuned for pelvic fracture diagnosis. In contrast, the ImageNet dataset was used to pre-train the Resnet101 backbone in the conventional transfer learning method, which doesn’t contain the characteristics of pelvic fracture. Hence, this method can significantly contribute to the improved diagnosis of pelvic fractures, leading to a reduction in morbidity and mortality. However, the evaluation of pelvic fracture detection performance was limited to a single deep convolutional neural network (DCNN) with different pre-training schemes. Given the unique characteristics of pelvic fractures, it is important to further evaluate the method using various types of DCNNs before considering practical implementation. Additionally, it is important to note that it was a retrospective study, and the data were from a single institute, which introduces the possibility of population bias. Moreover, the selection of PXR images and 3D-CT scans was performed randomly, potentially including selective bias. Therefore, the interpretation of the findings may differ when applied to other institutes or populations. Consequently, it is crucial to validate the proposed method using larger and more diverse datasets to establish its usefulness in different hospital settings.

## Methods

### Subjects and materials

The data were collected from a total of 478 subjects with a mean age of 64.22 ± 19.08 years. The range of age was from 20 to 93 years. Among the subjects, 268 were male and 209 were female. 3D-CT were acquired from 473 and 201 subjects had pelvic fractures. The CT images were acquired using multidetector-row CT (MDCT) scanners with a tube voltage of 120kVp and auto mAs. Additionally, a total of 481 PXR images were obtained from 315 subjects. Among the PXR images, 365 images from 199 subjects had fractures. All the data were obtained at Steel Memorial Hirohata Hospital in Japan between April 2013 and August 2019. The existence of fractures in 3D-CT and PXR images were confirmed by expert radiologist and doctors from Steel Memorial Hirohata Hospital, Japan.

### Synthesizing PXR images from 3D-CT using DRR

In this study, DRR^46^ volume rendering, also known as simulated x-ray (XR) rendering is used to synthesize PXR images from 3D-CT. This method involves simulating x-rays passing through a reconstructed CT volume by considering the tissues absorption properties. We created a parallel projection algorithm that can be explained by Eq. (1).

1 $${X}_{DRR}(i,j)= \frac{1}{N}{\sum }_{k=1}^{N}{e}^{\left(\frac{\alpha }{100}\right)\times \left(\frac{{X}_{CT}\left(i,j,k\right)+1024}{1000}\right)}$$

where α is the absorption coefficient, *X*_*CT*_ is the CT value in Hounsfield unit (HU), and *X*_*DRR*_ is the synthesized value. α controls the boosting of X-ray absorption as the tissue density increases. For this study, the value of α is chosen to be 90.

### DCNN training and fine-tuning

**Figure 8:**
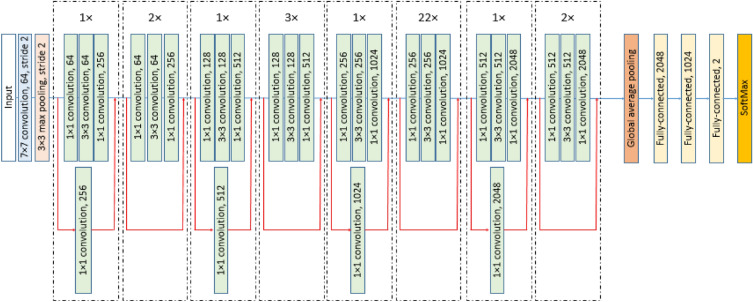
Architecture of DCNN.

In this study, a residual block-based architecture named Resnet101^47^ is utilized as the backbone of DCNN. The DCNN consists of residual blocks of Resnet101 followed by a global average pooling layer, three fully-connected (FC) layers, a softmax (SM) layer and a classification (CL) layer. The architecture of the DCNN is illustrated in Fig. 8. The backbone contains 8 residual blocks. Each of the residual blocks has 3 convolution layers followed by a batch normalization (BN) layer and a rectified liner unit (ReLU) layer. The input and output of the 2nd, 4th, 6th, and 8th residual blocks are added elementwise. Convolution operation and upscaling are performed on the input of the 1st, 3rd, 5th, and 7th residual blocks before adding elementwise to their respective output. The 2nd, 4th, 6th, and 8th convolution blocks are repeated 2, 3, 22, and 2 times, respectively. Categorical cross-entropy is used as the loss function with class weights to address the class imbalance. Equations (2)–(3) are used for calculating the class weights.

2 $${CW}_{F}=\frac{{n}_{N}}{{n}_{N}+{n}_{F}}$$

3 $${CW}_{N}=\frac{{n}_{F}}{{n}_{N}+{n}_{F}}$$

where *CW*_*F*_ is the class weight for fracture class, and *CW*_*N*_ is the class weight of normal class. *n*_*N*_ and *n*_*F*_ are the number of normal PXR images and the number of PXR images with fracture, respectively. All the processing and training is done using MATLAB 2022b (x64) on a computer with AMD Ryzen 7 2700 8-core processor (3.20 GHz), DDRAM 32 GB, and NVIDIA Titan RTX graphics card.

The synthesized PXR images and acquired PXR images differ in size. To standardize the input for the DCNN, all images are initially downsampled to a size of 224 × 224. Then the intensity values are linearly converted to the range of 0–255. Later, the training images are augmented using random rotation, translation and scaling. In the DRR-based method, the DCNN is first trained using augmented synthesized PXR images for 110 epochs, with a batch size of 98 and a regularization parameter of 0.00001. The initial learning rate is 0.00005 and decreased by a factor of 0.1 every 8 epochs. In the fine-tuning step, the FC, SM, and CL layers of the trained DCNN are re-trained using PXR images for 38 epochs. The initial learning rate is 0.0000005, and the regularization parameter is 0.00001. The batch size remains 98, and the learning rate is dropped by 0.1 every 10 epochs. The same hyper-parameters are used for fine-tuning the DCNN in the conventional ImageNet-based method.

When combining DRR-based method and ImageNet-based method in ImageNet + DRR20, and ImageNet + DRR20_Full training scheme, the DCNN initialized with ImageNet dataset is trained using synthesized XR images for 58 epochs, with a batch size of 98. The initial learning rate is set to 0.0000005, and the regularization parameter remains 0.00001. The learning rate is dropped by 0.1 every 10 epochs. The aforementioned hyper-parameters are used for fine-tuning in both the training schemes.

### Evaluation

To evaluate the performance of DCNN on a dataset, fivefold cross-validation is used. The AUROC as well as F1 score is calculated to compare the performance. Prior to plotting the ROC curve and F1 score, it is necessary to calculate sensitivity and specificity. Sensitivity and specificity are calculated using true positive (TP), false positive (FP), true negative (TN), and false negative (FN). TP represents the successfully detected PXR images with fractures, while FN represents the falsely detected PXR images with fractures. TN denotes the number of correctly detected normal PXR images. FN indicates the number of PXR images with fracture detected as PXR images without fracture. Sensitivity, specificity, and F1 score are defined by Eqs. (4)–(6). To calculate the F1 score, a confidence score threshold of 0.5 is utilized to determine TP, FP, TN and FN.

4 $$Sensitivity=\frac{TP}{TP+FN}$$

5 $$Specificity=\frac{TN}{TN+FP}$$

6 $$F1 score=\frac{2 \times Sensitivity\times Specificity}{Sensitivity+Specificity}$$

To plot the ROC curve, sensitivities and 1-specificities, also known as False positive rate (FPR), are calculated for various confidence score thresholds. Finally, AUROC is calculated by Eq. (7).

7 $$AUROC=\sum_{i=1}^{n}{sensitivity}_{i}\times {(FPR}_{i}-{FPR}_{i-1})$$

where *sensitivity*_*i*_ and *FPR*_*i*_ are the sensitivity and FPR, respectively, at the i-th point in ROC curve.

### Ethical approval

The Institutional Review Board of Steel Memorial Hirohata Hospital, Japan, granted ethical approval (IRB Number: 2019-1-52) for the study, and all analyses adhered to related regulations and guidelines. The requirement for informed consent from the study subjects was waived by the IRB of Steel Memorial Hirohata Hospital, Japan.

## Data Availability

The PXR and 3D-CT are not publicly available due to the restrictions of the policy of the Steel Memorial Hirohata Hospital, Japan. However, the datasets can be provided from the corresponding author with permission of the Steel Memorial Hirohata Hospital, Japan, on reasonable request.
